# Successful Pregnancy following Myomectomy Accompanied with Abdominal Radical Trachelectomy for an Infertile Woman with Early Cervical Cancer: A Case Report and Literature Review

**DOI:** 10.1155/2018/5623717

**Published:** 2018-07-02

**Authors:** Yuji Kamei, Ai Miyoshi, Nao Wakui, Takeya Hara, Serika Kanao, Hirokazu Naoi, Hirofumi Otsuka, Takeshi Yokoi

**Affiliations:** Department of Obstetrics and Gynecology, Kaizuka City Hospital, Kaizuka, Osaka, Japan

## Abstract

Women in the reproductive age group diagnosed with cervical cancer can receive radical trachelectomy in case they wish to preserve fertility. However, the indication for this procedure in infertile women with cervical cancer is controversial depending on the underlying cause of infertility. Here, we present a case of a successful pregnancy following myomectomy accompanied with abdominal radical trachelectomy for an infertile woman with early cervical cancer. The patient was a 38-year-old nulliparous woman with a significant past medical history of infertility of unknown origin. She had been undergoing treatment with assisted reproductive technologies including artificial insemination and in vitro fertilization for over four years. During her treatment for infertility, she was diagnosed with stage IB1 cervical squamous cell carcinoma. She received abdominal radical trachelectomy and abdominal myomectomy in the same surgical procedure. Six months after the surgery, she went for the first embryo transfer and became pregnant. At 26 weeks of pregnancy, a male baby weighing 980 g was delivered with an Apgar score of 3/5/7 by cesarean section due to chorioamnionitis. The baby has received general care in a neonatal intensive care unit for four months and weighed 4520 g when discharged.

## 1. Introduction

Radical abdominal hysterectomy and pelvic lymphadenectomy are usually performed for invasive cervical carcinoma. This surgical procedure results in the loss of fertility, and unfortunately, 40% of cervical cancer cases diagnosed in the United States occur in women of reproductive age [1].

Furthermore, there is an increasing number of women of advanced maternal age in developed countries. As such, the preservation of fertility has become a more important problem.

In cervical cancer stages IA2–IB1, women of reproductive age who desire preservation of fertility can receive radical trachelectomy. This procedure preserves the uterine corpus while resecting the cervix, parametrium, and upper part of the vagina. However, the indication for this procedure in infertile women with cervical cancer is debated; the most common causes of infertility are leiomyomas or uterine anomalies. To our knowledge, there is no report to date of a woman who underwent myomectomy for infertility and radical trachelectomy in the same procedure, who successfully became pregnant and delivered after the operation.

Here, we present a case of a successful pregnancy following myomectomy accompanied with abdominal radical trachelectomy for an infertile woman with early-stage cervical cancer.

## 2. Case Report

**Figure 1:**
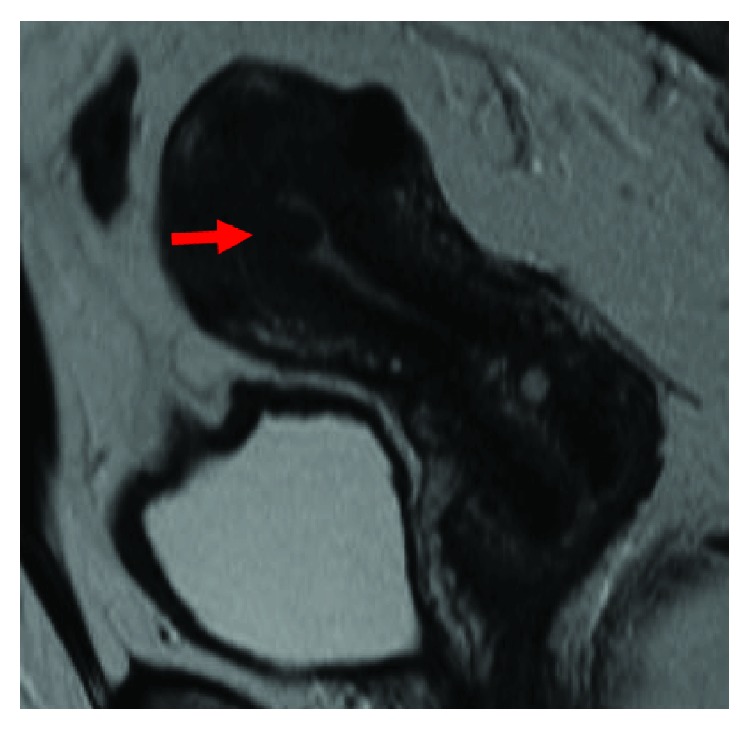
MRI image before operation; T2 weighted, sagittal section image. MRI did not describe the cervical mass, and a 15 mm myoma is located in the uterus (→).

The patient was a 38-year-old nulliparous woman who suffered from infertility of unknown origin. She had been treated with assisted reproductive technologies including artificial insemination and in vitro fertilization for over four years. During her treatment for infertility, cytological review followed by colposcopic biopsy revealed an invasive nonkeratinizing squamous cell carcinoma (SCC). A 1 cm mass was identified in the uterine cervix, but a pelvic MRI did not describe the cervical mass or parametrial invasion. Additionally, a submucosal leiomyoma of 15 mm in diameter was found in the uterus (Figure 1). CT scans showed no signs of lymph node swelling or distant metastases. Based on these findings, she was diagnosed with stage IB1 cervical squamous cell carcinoma. We offered radical hysterectomy and pelvic lymphadenectomy as standard treatment although she strongly desired fertility preservation. The submucosal leiomyoma may have been the cause of her infertility, and she was keen to resect the myoma during the same procedure. Submucosal leiomyomas can usually be resected with hysteroscopy but was not advised in this case from the oncological viewpoint. As such, we obtained informed consent and performed an abdominal radical trachelectomy followed by abdominal myomectomy.

During the surgery, we first drained the ascites in the pelvic cavity, resected bilateral pelvic lymph nodes, and sent them for intraoperative pathology. They were reported to be negative. The paravesical and pararectal spaces were then developed. The ureters on either side were resected to their insertion into the bladder. The uterine arteries were ligated and cut at the origin where they branched from the internal iliac arteries. Next, the uterosacral ligaments were divided. A colpotomy was performed circumferentially, and the cervical specimen was excised together with the parametrium at least 2 cm below the internal os. During the surgery, a frozen section procedure was performed for histology. The patient was found to have a 5 mm free cervical margin. A permanent cerclage was placed at the level of the isthmus. The uterus was then reanastomosed to the vagina. We then performed resection of the submucosal myoma via a uterine vertical incision. An intrauterine device (FD-1; Fuji Latex Co., Tokyo, Japan) was placed in the uterine cavity. The operation duration was 339 min, and blood loss was 500 ml. The surgery was completed with no complications.

The final histological specimen confirmed the diagnosis of squamous cell carcinoma, keratinizing type of cervix uteri, pT1B1. Exocervical, endocervical, and deep margin regions were negative. There was no metastatic lesion in the lymph nodes or lymphovascular space invasion. Leiomyoma of the corpus uteri showed no malignancy. No adjuvant treatment was administered, and no recurrence has been reported for at least 18 months postoperatively.

Six months after the surgery, she became pregnant following the postoperative first embryo transfer. The fetus was appropriate for gestational age. At 21 weeks of pregnancy, she claimed vaginal bleeding, and her lower uterine segment lengths were shortened from 23 mm to 13 mm. She was diagnosed with threatened abortion, and tocolysis was started. At 25 weeks, preterm premature rupture of membranes occurred. She received antibiotics, and intramuscular betamethasone was administered. At 26 weeks, a male baby weighing 980 g was delivered with an Apgar score 3/5/7 by caesarean section due to chorioamnionitis. The baby received general care in a neonatal intensive care unit for four months and weighed 4520 g when discharged. He is now 6 months old and is well. There has been no recurrent disease of her cervical cancer for 18 months from the trachelectomy and myomectomy.

## 3. Discussion

According to the SEER data, patients under the age of 45 account for approximately 40% of all cervical cancers [2].

As the childbearing age is increasing in Japan, the number of women diagnosed with cervical cancer on their reproductive age who wanted to preserve their fertility is on the rise. Therefore, the indication for radical trachelectomy should be widened, and more frequent problems of infertility before or after the surgery could arise.

Some authors reported that in the eligibility criteria for radical trachelectomy, clinical evidence of impaired fertility is not included [3, 4]. However, many reports showed that assisted reproductive technologies (ART) may allow some women suffering from infertility issues following radical trachelectomy to become pregnant [5, 6].

The upper age limit for radical trachelectomy tends to be older with the advance of ART. The upper limit age varies from 40 to 45 years old in most reports [3, 7], but a few reports did not limit the age for radical trachelectomy. Kay et al. reported a 45-year-old case of successful pregnancy following radical trachelectomy, ovum donation, and in vitro fertilization. This report suggested that radical trachelectomy can also be considered in a perimenopausal woman [8].

Until now, there has not been any report of an infertile woman receiving radical trachelectomy who became pregnant soon after the surgery. This is the first case report of a successful pregnancy in a previously infertile woman with cervical cancer, who underwent myomectomy for fertility treatment, accompanied with abdominal radical trachelectomy.

With regard to the radical trachelectomy, different procedures have been reported. The simple (type I) trachelectomy is typically limited to early-stage cervical cancer, stage IB1 or earlier disease, tumor size less than 2 cm, no lymphovascular space invasion, and no evidence of nodal metastasis. In simple trachelectomy, the parametrial tissue is not excised and the vaginal wall is incised circumferentially just above the cervix. Most reports commonly apply radical trachelectomy as an equally radical treatment as type III radical hysterectomy, which includes removal as far as possible from the uterosacral ligaments, parametrial resection as near as possible to the pelvic wall, and ligation of uterine vessels at the origin. However, the type III procedure may involve iatrogenic infertility with disruption of pelvic autonomic innervation [9]. The type II radical hysterectomy is an abbreviated procedure of type III, which preserves parametrial tissues and the ascending branches of the uterine arteries. Muraji et al. reported that modified (type II) radical trachelectomy applying type II radical hysterectomy is a feasible and safe operation [10]. However, the difference in oncologic outcome between type III and type II radical trachelectomy has not been fully discussed and should be further considered.

In fertility-sparing trachelectomy as in previous reports, improvements in surgical techniques allow for preservation of uterine arteries in most cases [3, 10–12]. On the other hand, some evidence indicates that the viability of the uterus can be maintained with only two infundibulopelvic ligaments, and live births have been reported in cases where the uterine arteries were not preserved [11, 13]. Tang et al. reported that preservation of uterine arteries is not necessary because anatomically preserved uterine arteries have a high chance of occlusion after the procedure [14]. It is still controversial as to whether the preservation of uterine arteries contribute to the pregnancy outcome.

Saso et al. reported that the overall pregnancy rate among the 61 patients who attempted to conceive after trachelectomy was 25% (24% in abdominal type III radical trachelectomy, 26% in abdominal type II radical trachelectomy, and 25% in abdominal type I trachelectomy) [15].

However, radical trachelectomy itself sometimes causes infertility following cervical stenosis. Cervical stenosis may impact infertility care because patients suffer from great changes of menstrual patterns or even amenorrhea [16]. Noyes et al. proposed the management of fertility issues such as cervical stenosis after radical trachelectomy. When the opening is pinpoint- or slit-like and small, dilation cannot be performed by standard gynecologic instrumentation. Instead, narrow dilators such as the Os Finder™ (Cooper Surgical, CT), a flexible, disposable size 2 French instrument made of a Teflon-like material, are used. For fertility evaluation and treatments, narrower malleable semirigid plastic alternatives, such as the Tom Cat™ (Tyco Healthcare, MA), can be utilized. This catheter can be used for hysterosalpingogram, IUI, and embryo transfer, if necessary. These techniques may also improve the pregnancy rate of infertile women after radical trachelectomy [1]. Okugawa et al. reported that 12 of 15 women experienced some infertility issues after radical vaginal trachelectomy conceived by ART [17]. They reported 4 of 6 cervical stenosis cases where pregnancy was achieved after cervical dilation and/or using ART. At present, cervical stenosis may be a problem with a viable solution.

Li et al. reported that postoperative cervical stenosis could be effectively prevented by installation of a tailed T-IUD during surgery [3]. Formerly, a catheter was placed in the cervical os, although the catheter dropped out soon after the surgery. We then installed an IUD into the uterus during radical trachelectomy, and we have not experienced cervical stenosis since. We think this method of installing an IUD is a simple and effective prevention for postoperative cervical stenosis.

In the present case, the patient's infertility issues had been investigated by an infertility specialist four years before the surgery, but the cause of infertility was unknown. She was treated with IVF-ET but could not conceive. In the perioperative investigation of cervical cancer, a submucosal leiomyoma of 15 mm was incidentally detected by MRI, and that was considered to be a potential cause of her infertility. We performed concurrent myomectomy with radical trachelectomy, and she conceived soon after the surgery. Hence, we assumed the cause of infertility was the failure of embryo implantation. This case suggests that uterine factors of infertility such as leiomyoma can be treated during radical trachelectomy. Another report documented one case of a concurrent hysteroscopic resection for a uterine septum successfully achieving pregnancy [6]. It may be permissible for us to perform hysteroscopy during the surgery, which can improve the pregnancy rate although there are few reports. In the reported case, no recurrence was observed for 12 months postoperatively.

The perinatal outcome of the present case was not favorable. Premature labor at 26 weeks occurred followed by pPROM. In the review of fertility-sparing surgery for cervical cancer [9], premature labor is as equally possible in abdominal radical trachelectomy as in vaginal radical trachelectomy. These days, all techniques aim to save at least 1 cm of cervical stroma, and this prevents ascending infection during pregnancy [9]. A short cervix or cervical incompetence is a known cause of pPROM and/or premature labor. Therefore, we also saved 2 cm of cervical length in the present patient.

Other risk factors for premature labor previously reported could be the removal of the lateral parametrium that may cause disruption of pelvic autonomic innervation [9]. Rob et al. reported that premature labor in abdominal radical trachelectomy is more likely than that in vaginal radical trachelectomy, where the uterine arteries are preserved and include a type II resection of the parametrium [9]. They hypothesize the cause of premature labor to be due to peritoneal adhesions or disruption of pelvic innervation.

In the present case, premature labor at 26 weeks occurred followed by pPROM, although we preserved at least 2 cm of cervical stroma. As such, sacrificing the uterine arteries and type III resection of the parametrium may affect the onset of premature labor.

No previous reports have showed that pregnancy with prior myomectomy increases the risk of pPROM. Therefore, we considered that the myomectomy did not affect the onset of pPROM in the present case. Even if we did not sacrifice the uterine arteries in type III resection of the parametrium and perform a myomectomy, the risk of premature labor in radical trachelectomy is still higher than the risk without the procedure. If the pregnancy had no complications, we planned to perform cesarean delivery at 36 weeks of gestation because we performed a vertical incision of the uterus during the myomectomy.

## 4. Conclusion

With advancements of ART, infertile woman with cervical cancer can receive radical trachelectomy should they desire preservation of fertility. We believe that radical trachelectomy could be performed for infertile women and that concurrent treatments for infertility should be performed during the radical surgery to improve pregnancy rates.
